# Strategies for Removal of Protein-Bound Uremic Toxins in Hemodialysis

**DOI:** 10.3390/toxins18010057

**Published:** 2026-01-22

**Authors:** Joost C. de Vries, João G. Brás, Geert M. de Vries, Jeroen C. Vollenbroek, Fokko P. Wieringa, Joachim Jankowski, Marianne C. Verhaar, Dimitrios Stamatialis, Rosalinde Masereeuw, Karin G. F. Gerritsen

**Affiliations:** 1 Department of Nephrology and Hypertension, University Medical Centre Utrecht, 3584 CX Utrecht, The Netherlands; 2 BIOS Lab on a Chip Group, Faculty of EEMCS, University of Twente, 7500 AE Enschede, The Netherlands; 3 IMEC the Netherlands—Health Research, 5656 AE Eindhoven, The Netherlands; 4 European Kidney Health Alliance (EKHA, WG3), 1000 Brussels, Belgium; 5 Aachen-Maastricht Institute for Cardiorenal Disease (AMICARE), University Hospital RWTH Aachen, 52074 Aachen, Germany; 6 Advanced Organ Bioengineering and Therapeutics, Faculty of Science and Technology, Technical Medical Centre, University of Twente, P.O. Box 217, 7500 AE Enschede, The Netherlands; 7 Division of Pharmacology, Utrecht Institute for Pharmaceutical Sciences, Utrecht University, 3584 CG Utrecht, The Netherlands

**Keywords:** protein-bound uremic toxins, renal replacement therapy, hemodialysis, adsorption, bioartificial kidney, tissue engineering

## Abstract

The removal of protein-bound uremic toxins (PBUTs) from the blood of kidney failure patients with conventional dialysis is limited. However, as their harmful effects and association with morbidity and mortality in dialysis patients are increasingly recognized, PBUTs have become important therapeutic targets. In this review, PBUT removal with current state-of-the-art dialysis technologies and future perspectives are discussed. Strategies to enhance PBUT clearance include methods that interfere with PBUT–albumin binding, such as chemical displacers, high ionic strength, pH changes, or electromagnetic fields, thereby increasing the free fraction available for dialysis. While these methods have shown promise in vitro, and some also in vivo, long-term safety data are lacking. PBUT removal can also be increased by adsorption, either directly via hemoperfusion, or indirectly, e.g., via sorbents incorporated in a mixed-matrix membrane or dissolved in the dialysate. In the kidney, PBUTs are secreted in the proximal tubules; hence, a cell-based bioartificial kidney (BAK) that secretes PBUTs is proposed as an add-on to current dialysis. Yet both PBUT adsorption strategies and, in particular, BAKs face considerable challenges in upscaling and mass production at acceptable costs. In conclusion, many novel technologies are under development, all requiring further (pre)clinical testing and upscaling before these strategies can be applied in the clinic.

## 1. Introduction

**Figure 1:**
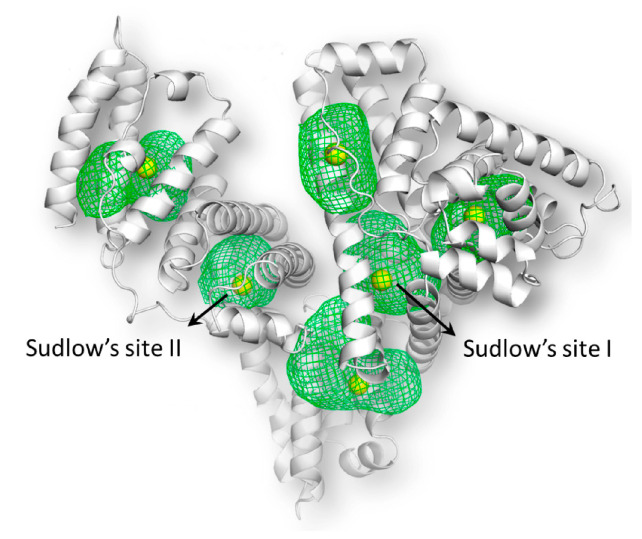
Human serum albumin and the two main PBUT binding sites. The green meshes represent the binding sites and the yellow spheres indicate their geometric center. Adapted from [10] (under a Creative Commons CC BY).

Kidney failure is characterized by the accumulation of uremic retention solutes. There are ongoing efforts to characterize and classify a growing list of over 140 solutes, a subset of which has shown harmful effects at high concentrations [1,2]. Among these are the so-called protein-bound uremic toxins (PBUTs), which comprise a class of low-molecular-weight compounds characterized by their high bound fraction to plasma proteins (Table 1), most notably albumin (Figure 1) [3,4,5]. Within this group are gut microbiota-derived metabolites, such as indoxyl sulfate (IS), indole-3-acetic acid (IAA), kynurenic acid (KA), p-cresyl sulfate (p-CS), p-cresyl glucuronide (p-CG), hippuric acid (HA), and 3-carboxy-4-methyl-5-propyl-2-furanpropionic acid (CMPF). IS and KA originate from tryptophan metabolism into indole, and p-CS and pCG from tyrosine metabolism into cresol. Indole and cresol are converted further into end-metabolites by the liver [6,7]. CMPF is derived from furan fatty acids, while HA is primarily derived from dietary glycine and benzoic acid [3,8,9].

In the kidney, only the free (unbound) fraction of PBUTs in plasma is filtered by the glomerulus, which limits their glomerular clearance. Instead, PBUT clearance relies to a large extent on active tubular secretion in the proximal tubule. This is facilitated by the basolaterally expressed organic anion transporters (OATs) 1 and 3, along with the apically expressed multidrug resistance proteins (MRPs) 2 and 4 and breast cancer resistance protein (BCRP) [11,12,13,14]. Conventional dialysis does not remove albumin, which is the main plasma protein, and lacks the tubular excretory function of native kidneys; hence, PBUT clearance is limited with dialysis. As a result, residual kidney function (RKF) remains an important route for PBUT excretion [15,16,17,18,19]. However, the near-absent RKF, together with poor dialytic removal of PBUTs, leads to considerable accumulation of these toxins in the plasma and tissues of dialysis patients, contributing to a.o. cardiovascular damage, progressive loss of RKF, and systemic inflammation [6,7,8,12,20,21,22,23,24,25,26]. In fact, the rise in plasma concentration of PBUTs in ESKD patients can reach up to 11-fold for p-CS and 43-fold for IS compared to healthy subjects, which is much higher than that of most free water-soluble low-molecular-weight toxins such as urea (~5-fold) [5]. This increase in PBUT concentration, in combination with their intrinsic toxicity, makes these toxins an important target for novel renal replacement technologies. However, although several association studies report that lower clearance or higher plasma levels of PBUTs are linked to increased morbidity and all-cause mortality—in some analyses independent of eGFR—[26,27], it is still unclear whether interventions that improve PBUT clearance have significant effects on hard endpoints.

**Table 1:** Overview of characteristics of several PBUTs in ESKD patients.

PBUT	Albumin Binding Site	Concentrations in Healthy Adults (mg/L) [28]	Concentration in ESKD (mg/L) [28]	Free Fraction	Dissociation Constant (M)	References
Indoxyl sulfate	Sudlow I/II	0.5 ± 4.0	37.1 ± 26.5	7.0–9.4%	Site I: 1.10 × 10^−3^ Site II: 3.10 × 10^−5^	[4,5]
p-Cresyl sulfate	Sudlow I/II	1.9 ± 2.3	23.0 ± 16.9	8.0–9.0%	Site I: Unknown Site II: 3.07 × 10^−4^	[4,5]
Indole-3-acetic acid	Sudlow I/II	17.5 ± 17.5	1004 ± 702	14.0–16.3%	Site I: 3.27 × 10^−3^ Site II: 6.72 × 10^−5^	[3,5]
Hippuric acid	Conflicting literature reports	3.0 ± 2.0	109.4 ± 64.7	50–60%	Site I: Unknown Site II: 7.8 × 10^−4^	[4,5,29]
3-Carboxy-4-methyl-5-propyl-2-furanpropionic acid	Sudlow I	4.6 ± 1.8	26.0 ± 10.2	<1%	Site I: 7.6 × 10^−8^	[30]
Kynurenic Acid	Sudlow I	(5.5 ± 1.3) × 10^−3^	(151 ± 76) × 10^−3^	29%	Site I: 9.7 × 10^−3^	[31,32]
p-Cresyl glucuronide	Unknown	0.03 ± 0.02 [33]	0.93 ± 0.60 [33]	79%	Unknown	[34]

In this review, we focus on various strategies aimed at improving PBUT removal. In short, these strategies can be grouped according to (a) dialysis modality or schedule, (b) increasing the free fraction of PBUTs, (c) sorbent techniques, and (d) biological approaches, namely the bioartificial kidney.

## 2. Dialysis Modality or Schedule

Conventional hemodialysis (HD), which is based primarily on diffusive transport, offers limited PBUT clearance due to their low free fraction. Hemodiafiltration (HDF) offers both diffusive and considerable convective transport. Theoretically, the additional convective component with HDF is expected to enhance the clearance of PBUTs. There is a limited number of studies comparing PBUT clearance between HDF and HD [15,35,36,37,38,39,40,41,42,43,44,45]. Regarding efficacy parameters such as clearance and reduction ratios (RRs), there does not appear to be a consistent effect of HDF versus HD (Supplementary Table S1). Among the nine studies comparing PBUT clearance or RR between HD and HDF, only Bammens et al. [35] reported a higher clearance of p-CS, and Cornelis et al. [37] reported significant improvements in RR for IS and IAA with postdilution HDF (pdHDF). In the long term, there was no consistent reduction in predialysis PBUT concentrations with HDF compared to HD [41,44], although Meert et al. [46] reported significantly decreased plasma concentrations of p-CS and CMPF after 9 weeks of treatment with pdHDF, and Panichi et al. [43] found decreased concentrations of IS and p-CS after 6 months of pdHDF treatment. Krieter et al. (2019) found a transient decrease in predialysis IS concentrations after 3 weeks, but this did not persist after 6 weeks [41]. In conclusion, the current, albeit limited, evidence does not support a clinically relevant beneficial effect of HDF as a standalone therapy compared to HD regarding PBUT removal. As an alternative, extended-hours dialysis, e.g., 8 h sessions, showed a significant improvement in p-CS removal over a single session [37]. This schedule allows for a rebalancing of the free and bound fraction of PBUTs, as well as equilibration between the extravascular compartment and the plasma, but again, no significant decrease in PBUT plasma concentration was found in the long term (after one year) [47].

Some authors have hypothesized that dialytic albumin loss may enhance PBUT clearance and thereby contribute to improved survival [48]. However, in a small prospective crossover study including 22 patients, increased dialysate albumin loss with medium cut-off membranes was not associated with greater removal of IS and p-CS [45].

## 3. Increasing the Free Fraction of PBUTs in Plasma

Negatively charged PBUTs bind to albumin, primarily at Sudlow sites I and II, which are locally positively charged regions. This binding occurs via hydrophobic interactions with protein residues, as well as electrostatic interactions with hydrophilic residues, mainly through hydrogen bonding (Table 1) [3,9,49,50]. Both sites are formed by a hydrophobic cavity with polar residues in the pocket and a hydrophilic mouth [49]. p-CS, IS, and IAA bind reversibly and with relatively high affinity to Sudlow site II. IS and p-CS also bind with lower affinity to Sudlow site I, which is the sole binding site for CMPF [9,22,50]. The high-affinity binding site of HA is currently unclear due to conflicting literature. Data from Tao at al. [29] indicate that the primary binding site of HA is Sudlow site I, because the site I displacer furosemide demonstrated greater HA displacement from albumin than the site II displacers ibuprofen and tryptophan. However, molecular docking and displacement studies performed by Zaidi et al. revealed that HA binds to both sites, but more strongly to site II [48].

Various methods of increasing the free fraction of PBUTs have been investigated (Supplementary Table S2a–c). They mainly aim to decrease protein–toxin binding and increase the toxin’s free fraction in plasma. A higher free fraction would result in a higher concentration gradient across the hemodialysis membrane, which would improve toxin removal. This can be achieved by (i) adding chemical displacers which compete for the albumin-binding sites, leading to release of the toxins, or (ii) by modifying the structure of albumin and/or interfering with the electrostatic protein–toxin interaction. The latter can be accomplished via increasing the plasma ionic strength (IPIS), altering the pH, or applying a strong electromagnetic (EM) field.

### 3.1. Use of Chemical Displacers

Chemical displacers are albumin-binding ligands themselves, which can either directly compete for binding sites (e.g., ibuprofen, furosemide, and tryptophan) or induce bound-ligand release through allosteric mechanisms such as conformational state transitions, which have different affinities for specific compounds [29,51]. The latter is the case for fatty acids, which, upon binding, induce the transition of albumin from the so-called ‘neutral state’ to the ‘basic state’, which involves conformational rearrangements, leading to altered ligand-binding properties. A possible implementation of this method is infusing the displacer directly upstream of the dialyzer, thereby inducing PBUT release from albumin and increasing the free fraction available for removal as blood traverses the dialyzer. The effectiveness of a chemical displacer depends on its affinity for the Sudlow sites or the displacer’s allosteric influence on them, as well as on the binding affinity of the respective PBUTs for those sites. Furthermore, to prevent accumulation it is important to consider whether displacers are mainly cleared by the kidney, such as ibuprofen and furosemide [52], or extrarenally (e.g., fatty acid metabolism by β-oxidation in the liver and other tissues [53]) (Table 2). See Supplementary Table S2a for details on displacers tested in the literature. Here, we review the most relevant studies and the available evidence in vitro and in vivo.

**Figure 2:**
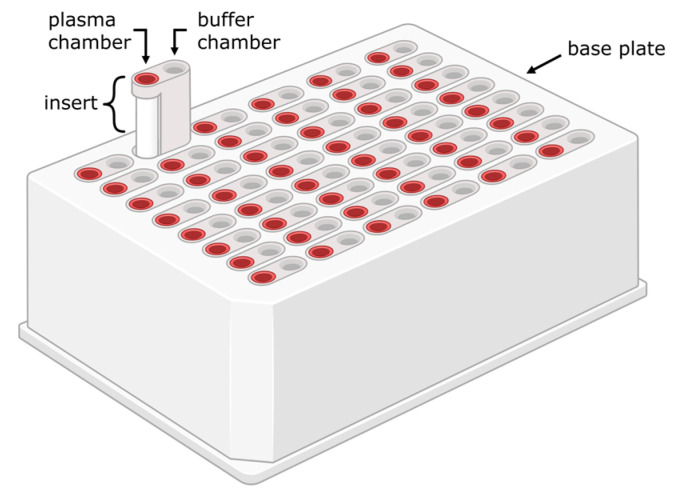
The rapid equilibrium dialysis (RED) device (Thermo Scientific, Waltham, MA, USA), available with 8 kDa or 12 kDa MWCO membranes. Created in BioRender. Brás, J. (2026) https://BioRender.com/8imi8oo.

Static experiments with displacers have been performed using free fatty acids (FFAs–a.o. oleic, linoleic, and caprylic acid), ibuprofen, furosemide, and tryptophan. Using either a ‘rapid equilibrium dialysis’ approach (Figure 2) or ultrafiltration devices for the measurement of protein binding, these displacers showed a significant increase in the free fraction of several PBUTs (CMPF, p-CS, IS, and/or 3-IAA), resulting in enhanced removal (Supplementary Table S2a) [29,30,54,55,56]. Interestingly, a synergistic effect of ibuprofen and furosemide on IS removal was observed [29]. HA was also displaced from human serum albumin (HSA) by ibuprofen and furosemide, but not by tryptophan.

Various studies reported on in vitro (hemo)dialysis models using either whole blood or an albumin solution spiked with uremic toxins and single-pass dialysate flow. Infusion of ibuprofen and furosemide to albumin upstream of the dialyzer increased IS and IAA removal, with minimal effect on HA (Supplementary Table S2a) [29]. Of note, furosemide was used at a plasma concentration of 1 mM, exceeding the levels at which toxicity has been reported (>0.25 mM [57]). In addition, infusion of tryptophan and docosahexaenoic acid (DHA) increased IS removal, with tryptophan showing the least effect and DHA showing the greatest enhancement, up to 2.7-fold [58]. Similarly, Shi et al. (2022) found an increase in p-CS, IS, and IAA removal with FFA infusion [59]. More recently, Raillon et al. tested various fatty acids with in vitro hemodialysis using spiked bovine blood. Capric acid (decanoate; C10) and caprylic acid (octanoate; C8) demonstrated the highest displacement effect, with octanoate infusion producing a steady-state plasma concentration of 1 mM that increased the fractional removal of IS and p-CS by 2.5-fold and 2.3-fold, respectively [56].

Several studies examined the effect of displacers on PBUT removal in a 5/6th nephrectomy rodent model. Li et al. (2019) found an increase of up to 3.7-fold in dialysis efficiency of IS and p-CS using salvianolic acids, which are water-soluble polyphenolic compounds with anti-oxidant properties (Supplementary Table S2a) [60]. However, this study employed microdialysis at a dialysate flow not scaled to body weight which makes an accurate and meaningful interpretation of the results difficult. Intravenous administration of an FFA mixture of oleic and linoleic acid during 3 h hemodialysis with body weight-scaled flows (blood flow 1 mL/min, dialysate flow 5 mL/min) increased total solute removal (TSR) of p-CS, IS, and IAA up to 4.6-fold [61]. In another study, the same group observed significant improvements in the RR of p-CS, IS, and IAA, but not of HA, with either intravenous FFA infusion or human serum albumin addition to the dialysate, with the largest effect occurring when both strategies were combined (Supplementary Table S2a) [55].

A single clinical study in hemodialysis patients (*n* = 18) without residual diuresis showed that ibuprofen infusion upstream of the dialyzer at a time-averaged median blood concentration of 0.16 mM significantly increased IS and p-CS clearance by ±2.4-fold, with levels returning to baseline post-infusion [62]. Thus, sustained infusion appears necessary to maintain clearance. No safety data were reported. In addition, a similar study will start in the near future to evaluate the effectiveness of infusing lipid emulsions during hemodialysis [63,64].

**Table 2:** Overview of chemical displacers.

Displacer	Primary Albumin Binding Site	Elimination Pathway	References
Ibuprofen	Site II	Metabolized in the liver into inactive metabolites, which are excreted in urine	[65]
Furosemide	Site I	Minimal hepatic metabolism; primarily excreted unchanged in urine	[52]
Tryptophan	Site II	Metabolized in the liver; most metabolites, including several PBUTs, are excreted in urine	[66]
Salvianolic acids	Site II	Metabolized in the liver; most metabolites are excreted in urine	[67]
Free fatty acids	Site II	β-oxidation in mitochondria, primarily in the liver	[68]

In summary, displacers like FFAs and certain drugs can enhance PBUT clearance during dialysis, with promising in vitro and preclinical results, although there are safety concerns regarding some compounds or their concentrations and more clinical evidence is needed. Combining displacers—potentially at lower, safer doses—that target different binding sites may represent a rational next step toward a more effective, safer and potentially clinically viable displacement-based PBUT removal therapy, particularly given that various PBUTs bind to both Sudlow sites (see Table 1) such that blocking one site may still leave the other accessible.

### 3.2. Increased Plasma Ionic Strength (IPIS)

The ionic strength of blood plasma can be enhanced by increasing the sodium (Na^+^) concentration, which alters the electrostatic interactions between albumin and PBUTs, and results in an increased toxin free fraction [50]. In vitro studies have shown that the free fractions of IS, p-CS, and IAA increase linearly with sodium concentration (up to 2.5-, 1.9-, and 1.2-fold, respectively, at 500 mM compared to 150 mM) in albumin solutions and both healthy and uremic plasma (Supplementary Table S2b) [22,30]. However, this did not result in an increase in CMPF removal in static rapid equilibrium dialysis [30]. In in vitro predilution HDF using human blood and applying a sodium concentration of 500 mM, the larger free fraction of IS (2.8-fold increase) and p-CS (3.0-fold increase) resulted in a significant 1.7-fold and 1.5-fold increase in the clearance of IS and p-CS, respectively [69]. Importantly, increased plasma ionic strength (IPIS) did not alter the activity of the biomarkers LDH, superoxide dismutase, or alkaline phosphatase in vitro, suggesting that IPIS does not affect blood cell survival. Furthermore, the sodium concentration at the dialyzer outlet was similar in isotonic and hypertonic dialysis, using a dialysate sodium concentration of 130 mM during hypertonic dialysis. In prevalent HD patients (*n* = 8), HDF with IPIS ([Na^+^] 240 mM) showed a moderate, non-significant increase in p-CS RR and clearance compared to HD and conventional HDF [38]. For IS, a more pronounced effect was observed, but only the clearance of free IS was significantly higher with HDF at IPIS compared to conventional HDF and HD. The plasma sodium concentration at the dialyzer outlet was 4 mM higher than at the inlet using a dialysate sodium concentration of 130 mM, and the total sodium removal was considerably lower compared to HD and HDF, but this did not result in a higher interdialytic weight gain. Hemolysis, a potential side effect due to the rapid changes in osmolality, was negligible ex vivo ([Na^+^] 500 mM), in vivo in a sheep model ([Na^+^] 600 mM), and in patients ([Na^+^] 240 mM). Other adverse events, such as hypo- or hypertension or clotting of the extracorporeal circuit, were not observed.

Overall, while IPIS appears technically feasible and safe in short-term experimental and small clinical studies, larger and adequately powered clinical trials are required to determine its long-term effects on plasma PBUT concentrations, sodium balance, and hemocompatibility. Further optimization of dialysate sodium concentrations (<130 mM) is also needed. The current lack of commercially available low-sodium dialysate, together with the need for a more complex and costly hemodialysis setup using additional filters and pumps, may limit widespread clinical implementation.

### 3.3. Changing the pH

Changes in pH influence protein folding and net charge, and, consequently, toxin–protein interaction (Supplementary Table S2c). For instance, HSA can reversibly change from its regular heart-shaped conformation at physiological pH (7.4) to an extended conformation below pH 2.7 [51]. In addition, a change in pH from 7.4 to 4.0 significantly alters the protein’s net charge from negative (−19) to positive (+30), thereby impacting electrostatic interactions [51,70]. In serum of uremic patients, Yamamoto et al. (2021) found an increased free fraction of various PBUTs at pH 3.2 (IS 5.3-fold, p-CS 3.9-fold, IAA 3.3-fold) and pH 11.3 (IS 4.2-fold, p-CS 3.8-fold, IAA 4.2-fold) compared to ‘near-neutral’ pH 8.2 [71]. This pH range is far from (patho-)physiological levels (7.0–7.8), however [72]. Over a smaller pH range (6.0–8.5), Shi et al. (2019) did not observe significant effects in static rapid equilibrium dialysis [30]. Neither study examined biocompatibility or safety, such as the occurrence of hemolysis, the effects on enzyme function, or if dialyzer outlet pH was outside of the physiological range, which is essential for future clinical application.

### 3.4. Electromagnetic Field/Electrical Current

The use of EM fields is reported to enhance dissociation of PBUTs from albumin, either via conformational changes of albumin or via disruption of electrostatic interactions between PBUTs and albumin [73,74]. Klos-Witkowska et al. found that exposure of bovine serum albumin (BSA) to EM fields with frequencies of 125, 180, and 230 MHz resulted in changes in UV/Vis spectrophotometry absorption intensity, suggesting conformational changes of albumin and increased exposure of amino acid residues to the environment [74]. A study examining the interaction between BSA and norfloxacin (NRF)—an antibacterial agent whose primary binding site is Sudlow site I [75]—found that exposing NRF-BSA complexes to RF waves in the frequency band of 1 Hz–1 MHz resulted in a near six-fold decrease in affinity, possibly due to a change in the structure of albumin [76]. Initiatives studying the effects of EM fields on PBUT–protein interaction are at an early stage of development. Jankowski et al. patented a device that applies high-frequency EM fields to disrupt PBUT–protein interactions using two parallel plate electrodes surrounding the dialyzer [77]. Frequencies in the range of 1–20 MHz increased dialytic removal of phenylacetic acid 1.3-fold and IS 1.4-fold. In the 110–115 MHz range, an increase in removal of 1.3-fold was observed for phenylacetic acid, but no data was reported for IS. Further confirmation of PBUT removal using EM waves and elucidation of the mechanism of action and reversibility of protein conformation changes after EM wave exposure is required. In addition, miniaturization of such a setup should be realized for better integration with current dialysis methods, and, in particular, electromagnetic interference of other electronic devices must be avoided. Finally, the safety of the method is yet to be evaluated [77].

## 4. Sorbent Techniques

There are four distinct sorbent-based HD strategies for improving PBUT removal: sorbent-containing dialysis membranes, the use of sorbents in the dialysate (either as a cartridge or dissolved in the dialysate), direct hemoperfusion over a sorbent, and finally ‘fractionated plasma separation, adsorption, and dialysis’ (FPAD) systems. The first two strategies are based on increasing the concentration gradient of the free PBUT fraction across the dialysis membrane [78], while the last two are based on direct removal of PBUTs from the albumin.

### 4.1. Sorbent-Containing Dialysis Membranes

**Figure 3:**
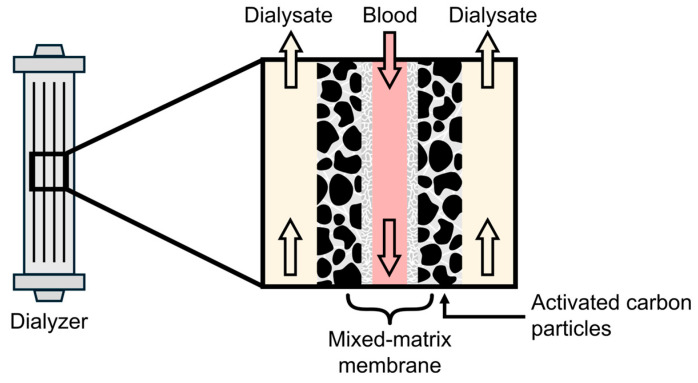
Mixed-matrix membrane (MMM) containing activated carbon particles. The large arrows represent the fluid flow direction.

Adding sorbents to the dialysis membrane has primarily been examined in the form of so-called dual-layer mixed-matrix membranes (MMMs). These consist of a thin, porous, hemocompatible, and particle-free filtration layer akin to conventional dialysis membranes, surrounded by a second layer containing activated-carbon (AC) sorbent particles incorporated in a macroporous polymer matrix (Figure 3). This way, PBUTs that diffuse over the filtration layer are adsorbed instead of entering the dialysate, which maximizes the concentration gradient within the membrane [78]. In vitro results of HD using an MMM dialyzer and human plasma showed a 1.4- to 4.2-fold increase in total solute removal of IS compared to conventional dialyzers (Supplementary Table S3) [79,80,81], and a 1.7-fold increase in p-CS TSR [81]. Results for HA varied from equal TSR [79] to a 1.7-fold increase in TSR compared to conventional dialyzers [80]. The dual-layer MMMs showed good hemocompatibility [82]. Of note, all experiments were conducted with dialysate recirculation, whereas standard hemodialysis employs single-pass dialysate. This experimental setup may inadvertently confer a systematic advantage to mixed-matrix membranes over conventional dialyzers, as the adsorption of toxins in the membrane’s outer layer prevents their accumulation in the dialysate, whereas with standard dialyzers, toxin buildup in the dialysate progressively reduces the diffusion gradient during the experiment. Ter Beek et al. addressed this issue by comparing solute dialysance [83].

### 4.2. Sorbents in the Dialysate

PBUT sorbents can also be added to the dialysate. This technique doubled the in vitro clearance of p-CS with AC in the dialysate compared to standard dialysate [84]. In fact, the clearance of this solute with AC added to the dialysate and with a 12-fold reduction in dialysate flow rate was similar to standard dialysate at regular flow rates. In an in vitro model of HD using human plasma, Li et al. showed increased removal of IS (1.9-fold), HA (1.3-fold), and p-CS (1.5-fold) by adding poly-cyclodextrins to the dialysate [85,86]. In a static binding assay, liposomes at the dialysate side showed higher binding of IS, IAA, and p-CS compared to standard dialysate and an albumin solution. Liposomes are spherical and hollow sub-micrometer-scale structures made up of a phospholipid bilayer, which predominantly exert hydrophobic interactions with PBUTs [59]. The difference was highest for cationic liposomes, which is likely a result of the net negative charge of PBUTs, indicating that electrostatic interactions also contribute to the adsorption [55]. The same effect was observed in an in vitro HD experiment, although with a lower difference in PBUT removal between liposomes and albumin [61,87]. In vivo, addition of either albumin [59,61,87] or liposomes [87] to dialysate yielded significantly higher RRs and clearance rates for p-CS, IS, and IAA, but not for urea, creatinine, and HA.

### 4.3. FPAD and Hemofiltrate-Reinfusion Systems

**Figure 4:**
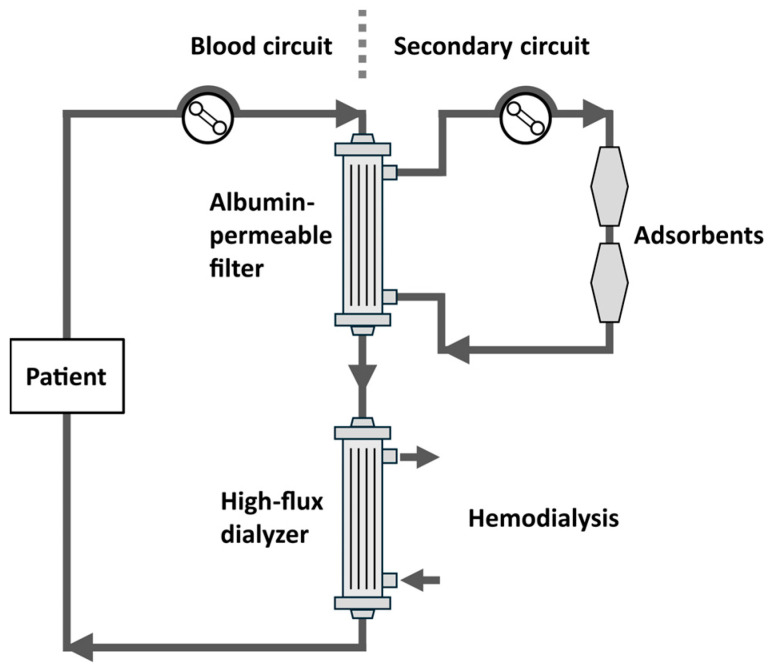
Fractionated plasma separation, adsorption, and dialysis (FPAD) system.

The so-called fractionated plasma separation, adsorption, and dialysis (FPAD) system includes a plasma filtration unit positioned up- or downstream of the dialyzer as shown in Figure 4. A plasma filter or plasma separator separates plasma from whole blood while retaining cellular components. The filtered plasma (containing plasma proteins) is subsequently circulated through a secondary circuit containing a sorbent unit, which adsorbs the uremic toxins, including PBUTs, after which the plasma is returned to the circulation.

Two clinical studies in stable ESKD patients on conventional HD were performed with FPAD. Using the Prometheus system (Fresenius Medical Care, Bad Homburg, Germany), Meijers et al. showed a ±2-fold increase in the RR of p-CS, as compared to high-flux HD. However, thrombotic complications occurred in three of the four patients, probably due to nonspecific adsorption of anti-coagulant factors, which led to premature termination of the study [88]. Brettschneider et al. (2013) examined two parallel cohorts of five patients on FPAD and high-flux HD [89]. While no RRs over the total session or clearance data were provided, FPAD demonstrated 1.8-fold and 2.0-fold greater reductions in IS and p-CS, respectively, over the extracorporeal circuit compared to high-flux HD.

Riccio et al. performed a clinical study in 12 patients with a hemofiltration–reinfusion (HFR) setup using a double-chamber HDF system in which the ultrafiltrate returns to the patient after its regeneration through a resin cartridge that binds hydrophobic and protein-bound solutes. This study found a 1.4-fold improvement in the RR for p-cresol after 4 h of treatment as compared to high-flux HD [90].

### 4.4. Hemoperfusion

Hemoperfusion is a blood purification technique where a patient’s blood is passed through a column containing adsorbent material such as activated charcoal, inorganic aluminosilicates (zeolites), ion exchange resins, or synthetic polymers. This strategy should theoretically be one of the most effective options to remove toxins, but early iterations suffered from hemocompatibility problems such as leukopenia and thrombocytopenia, which prevented clinical use [91,92,93,94]. Additionally, charcoal hemoperfusion has been linked to an increased risk of bleeding due to impaired platelet aggregation and enhanced fibrinolysis [95]. Other side effects include hypocalcemia, hypoglycemia, and neutropenia [93].

These side effects may be mitigated by coating the adsorbent material with polymer solutions, such as polyvinylpyrrolidone (PVP), which reduce platelet adhesion and complement activation. In dynamic experiments, PVP-coated activated charcoal (PVP-AC) demonstrated high toxin adsorption, achieving 39 mg/g of IS and 42 mg/g p-CS in the first hour [96], although comparison to conventional HD was lacking. PVP-AC beads showed better hemocompatibility in vitro compared to two commercial adsorber columns (DALI sorption system (polyacrylic acid-coated polyacrylamide beads, Fresenius) and Prismaflex Adsorba C300 (cellulose-coated activated charcoal, Gambro (now part of Baxter, Deerfield, IL, USA))) [97,98]. No significant change in biomarkers for activation of complement cascades, coagulation, or thrombocytes was observed. Leukocyte count was reduced by ±30%, as was the case with the DALI system [96]. Schildboeck et al. (2024) tested three commercial hemoperfusion adsorbers: Jafron HA (styrene-divinylbenzene copolymer, Jafron Biomedical Co., Zhuhai, China), Biosky MG (polystyrene resin, Biosun Medical Technology Co., Foshan, China), and Cytosorb (PVP-coated styrene-divinylbenzene copolymer, CytoSorbents Corporation, Princeton, NJ, USA) [99]. Although static whole-blood experiments showed significant removal of IS, HA, homocysteine, tryptophan, and CMPF, hemoperfusion did not outperform HD in dynamic in vitro tests. Zeolite silicalite and nitrogen-doped porous carbon adsorbents demonstrated a high PBUT-binding capacity of up to 106 mg/g for p-cresol in static experiments [100,101], but potential aluminum leaching from zeolites is a concern.

Yamamoto et al. (2018) performed a small clinical study in 15 stable HD patients with Lixelle S-35 (Kaneka Corporation, Osaka, Japan), a hemoperfusion column containing immobilized cellulose beads with a safe track record [102,103,104]. Although in vitro results showed promising PBUT adsorption, the addition of the Lixelle column upstream of a conventional dialyzer offered no significant improvement in IS, IAA, and p-CS removal compared to conventional HD.

In conclusion, while hemoperfusion adsorbents demonstrate in vitro potential for PBUT removal, their added clinical value over conventional HD alone remains uncertain regarding efficacy and safety. Further research is needed to establish their clinical relevance and long-term outcomes.

## 5. Bioartificial Kidney

Native kidneys clear PBUTs very efficiently, mainly via secretion by proximal tubular epithelial cells (PTECs) [11,12]. Cell-based modules, so-called bioartificial kidneys (BAKs), are under development using PTECs coated on membranes [51,105,106], with the aim of replacing tubular function, as current dialysis only replaces the filter function of the glomeruli. While recent advances have been reviewed in-depth elsewhere [107], here we provide a short overview of PBUT removal by BAKs.

In 1999, Humes et al. reported on OAT-mediated secretion of para-aminohippuric acid in a BAK consisting of porcine PTECs [108]. Although not reporting on PBUT removal, a small clinical trial with this BAK added to continuous renal replacement therapy (CRRT) in a cohort of 10 critically ill patients with multi-organ failure demonstrated the feasibility of the device in a clinical setting and suggested an immunomodulatory effect, with a less pro-inflammatory cytokine profile observed in some patients after treatment [109]. Building on this approach, Tumlin et al. conducted a randomized multicenter trial in 58 patients with acute renal failure requiring CRRT, showing a trend toward lower 28-day mortality (primary endpoint) with renal tubule cell therapy compared with CRRT alone (33% vs. 61%, *p* = 0.08). This was accompanied by a significant survival benefit over 180 days, with an approximately 50% reduction in mortality risk, as well as higher rates of renal recovery at 28 days (53% vs. 28%) as secondary outcomes [110].

More recently, Chevtchik et al. developed a BAK device for small-scale in vitro studies using OAT1-overexpressing human conditionally immortalized PTECs (ciPTECs), which were seeded on hollow fibers [105,106]. ciPTECs coated on these fibers were shown to be capable of active trans-epithelial transport of both anionic (IS [106] and KA [111]) and cationic compounds [105]. An IS clearance of ~50 μL·min^−1^·cm^−2^ was reported using a single ciPTEC-coated hollow-fiber membrane [111], amounting to a ~25-fold increase in clearance compared to conventional HD, adjusted for membrane area [112,113]. The transport of organic anionic compounds is facilitated by the presence of albumin [111]; therefore, modifications to this protein in CKD may also impact the transport efficiency in a BAK versus healthy albumin [114].

For cell-based renal replacement therapies, (testing of) biocompatibility may have unique challenges, even for single-use BAKs. For the ciPTEC-coated hollow-fiber membranes, it was shown that the cells did not secrete immune and inflammatory mediators to the blood side (only to the filtrate side after a stimulus) and had neither immunogenic nor tumorigenic and oncogenic characteristics, and remained functional in experimental dialysis conditions [106,115,116,117].

In silico models are also under development, which aim to characterize the transport kinetics of PBUT transporters in PTECs in order to support the design of future BAKs. These models have highlighted the impact of PBUT transporter density in the cultured cells and decreased transporter–albumin binding, which may result from CKD-modified albumin [118,119].

Recently, efforts have expanded toward implantable BAK devices, which aim to provide continuous, physiologically relevant toxin clearance, including PBUTs, without the need for external equipment. Kim et al. described an implantable bioreactor integrating human PTECs within a microfabricated device connected in series with an implantable hemofilter [120]. Similarly, the European KIDNEW project pursues the same goal [121].

## 6. Discussion and Future Outlook

Conventional dialysis provides insufficient removal of PBUTs, resulting in increased PBUT concentrations in the blood of ESKD patients, which is associated with increased morbidity, particularly cardiovascular. In this paper, we discussed multiple technologies under development to increase the removal of PBUTs by hemodialysis (See Table 3).

While HDF could theoretically increase the removal of PBUTs through convection—in particular those with a lower degree of protein binding such as HA—clinical results are disappointing. Importantly, though some short-term studies found slight improvements in terms of dialysis efficacy, HDF appears to have only limited to no effect on PBUT concentrations in the long term [15,43,44,46]. A possible explanation is that the low free fraction is still the rate-limiting factor for dialytic removal [8]. Slow dissociation of the protein-bound fraction, resulting in a decrease in the free fraction during dialysis, and a slow equilibrium between extravascular tissue and plasma may limit transport in the later stages of the session. Indeed, with conventional HD, protein binding of PBUTs was increased after 4 h of dialysis compared to the start of treatment [122]. Alternatively, as the PBUT free fraction is dependent on the available binding sites in serum albumin [24], lowering the total concentration of PBUTs whilst simultaneously increasing albumin concentrations (hemoconcentration due to ultrafiltration) might also contribute to the observed higher protein-bound fraction. Indeed, prolonging HD treatment time to 8 h allows for an equilibration between the extravascular compartment and the plasma, resulting in a large increase in total PBUT removal [37], although this also did not result in long-term beneficial effects on plasma PBUT concentrations [47]. As such, using HDF or extended HD specifically for improving PBUT removal is—at this time—not supported by evidence in the literature [15,44].

Displacement of the protein-bound fraction is a promising strategy, where in silico models showed a higher efficacy than HDF or sorbents in MMMs/in the dialysate [123]. In vitro and in vivo, various compounds increased the free fraction and clearance of PBUTs, with the largest effects being observed with FFA mixtures. Disruption of electrostatic interactions between PBUTs and albumin by increasing the NaCl concentration in the dialyzer also increased free fractions of PBUTs, with IPIS showing superior and more reproducible results compared to changing pH, which only showed an effect with large non-physiological pH changes [51]. However, long-term in vivo data on the use of displacers and IPIS are generally lacking, and suitability for chronic treatment is unclear at this time [124,125]. Displacers should ideally have a short half-life and be excreted through nonrenal pathways to minimize systemic accumulation and potential toxicity, both from the displacer itself and from the increased free fraction of PBUTs in systemic circulation. Ibuprofen has a short half-life of ~2 h [126,127] and is largely metabolized by the liver, but its metabolites are renally excreted and retain some activity [65]. Long-term treatment may therefore carry a risk of peptic ulcers and negatively impact residual kidney function [29]. Furosemide is mainly metabolized by the kidneys, leading to a long half-life in ESKD patients of up to 24 h [52,127,128]. Its accumulation may result in ototoxicity [129]. The long-term safety of FFA infusion as a displacer is unknown, but FFAs have a short half-life of 2–4 min [130], and intravenous lipid emulsions used in parenteral nutrition, which have a high oleic/linoleic acid content, are well tolerated and therefore appear to be a viable method [131,132]. Finally, it should be noted that using a chemical displacer may also benefit patients on peritoneal dialysis, which would ideally require a drug that is suitable for oral administration, while most displacers described here are for intravenous administration. IPIS may lead to increased sodium load and consequently an increased interdialytic weight gain and hypertension, although the net effect on plasma sodium can be prevented by using a lower-sodium dialysate. Further optimization of IPIS is needed to ensure complete removal of the excess infused sodium, since dysnatremia is a risk factor already present in ESKD patients [133]. Sodium removal also requires a more complex and costly hemodialysis setup that uses additional filters and pumps, and may therefore limit its widespread application in the clinic.

Sorbents can be applied via a less direct route of blood/body contact, i.e., in the dialysate and in the outer layer of the dialysis membrane, and thus, could potentially have less adverse effects compared to displacers. MMMs and sorbents in the dialysate appear more effective for IS and p-CS than for HA, likely because sorbents offer less additional benefit for small, weakly bound, less-hydrophobic solutes like HA, which already diffuse more readily through standard membranes. However, as with HDF and extended-hours HD, only the free fraction of PBUTs is cleared with sorbents at the dialysate side. This comes with all the previously mentioned limitations: slow dissociation of the protein-bound fraction, slow equilibration with the extravascular space, and an increase in the bound fraction during dialysis. A more direct means of adsorption, i.e., direct hemoperfusion, might theoretically achieve a significantly higher PBUT removal, though it requires sorbents with excellent hemocompatibility. Hemoperfusion cartridges are already commercially available and used in clinical practice for maintenance HD, primarily in Asia [134], though their added clinical benefit in PBUT removal remains uncertain [91].

Aside from potentially improving PBUT clearance, cell-based techniques may provide additional benefits due to the added tubular function, such as the reabsorption of useful solutes such as amino acids, thereby reducing the incidence of protein wasting, which is a major problem in dialysis patients [135]. In addition, these techniques may provide endocrine function in the form of 25-hydroxyvitamin D activation, given that vitamin D deficiency is prevalent in ESKD [108]. Of note, the cellular unit itself has no filtration capacity; thus, a dialyzer is still needed for the bulk of small-solute removal. BAKs would, therefore, be used as an add-on to conventional dialysis. Furthermore, these cell-based devices are yet to be established as safe and effective devices, while other future challenges, such as upscaling and mass production at low costs and off-the-shelf availability at dialysis units, must be addressed before clinical implementation.

Besides the use of the different strategies as standalone therapies, an interesting, and in the short-term feasible, next step would be a combination of strategies to further enhance the overall efficacy. Combinations may allow the use of lower concentrations or intensities of each strategy compared to their individual application to achieve a similar effect, therefore reducing adverse effects. One promising example is that of combining a technique that increases the free fraction, such as chemical displacers, with adsorbents during HD treatment. In uremic rats, the combined treatment of albumin as an adsorbent in the dialysate and the infusion of an FFA mixture at the blood side had a superior reduction rate of the measured PBUTs compared to either adsorbent or displacer individually [61,87]. However, the optimal combination of different strategies and specific interventions has yet to be determined.

It should be noted that most novel PBUT removal strategies are still in or just beyond the proof-of-concept phase, with low technology readiness levels (TRLs), and are clinically unproven. Lack of efficacy in vivo, potential safety issues such as hemocompatibility issues or adverse side effects, and finally, upscaling/manufacturing issues may limit the feasibility and preclude clinical application of these techniques. More importantly, even if proven safe and effective, it is not known whether improved PBUT clearance and sustained reduction in PBUT concentrations would result in improved patient welfare and/ or decreased morbidity and mortality. Notably, increased PBUT clearance does not necessarily result in lower plasma concentrations. In a crossover study, Camacho et al. showed that increased p-CS clearance by applying a combination of high dialysate flow and a dialyzer with higher flux (and surface area) did not lower p-CS plasma concentrations, which was due to increased p-CS generation during the higher clearance period [136]. Thus, reducing PBUT generation may also be important. Finally, the limited cost-effectiveness of the novel methods may hinder their widespread clinical use, especially in low- and middle-income countries. Moreover, present reimbursement criteria mainly lean upon Kt/V criteria for urea removal. This implies that—from a business perspective—the investment case for improved PBUT clearance technologies is not very attractive. To raise the TRL of a medical device prototype to a market-approved product requires many millions of dollars from investors, who (logically) demand good prospects regarding return on investment [137]. A prospect of increased reimbursement upon reaching certain PBUT clearance criteria might help. But regardless, it will remain the case that to become attractive for investors, technology for improved PBUT clearance should not only be medically advantageous and low-risk, but also cheap to implement and preferably seamlessly combinable with all existing HD systems, while requiring minimal schooling of staff.

**Table 3:** Overview of PBUT Removal Strategies.

Strategy	Mode of Action	TRL	Tested In Vivo	Clinical Evidence	Expected Increase in PBUT Clearance	Biocompatibility Concerns	Advantages	Limitations
Hemodiafiltration (HDF)	Diffusive and convective transport of PBUTs	9	Yes	Yes	+/−	None	Already used in clinical practice	More resource-intensive than standard HD, effect on PBUT removal uncertain
Chemical Displacers	Displacement of PBUTs from albumin	4–5	Yes	Yes	++	Moderate	Easily integrated into conventional HD	Risk of systemic toxicity, may affect other protein-bound drugs, sustained infusion needed
Increased Plasma Ionic Strength (IPIS)	Modification of PBUT–protein binding	3–5	Yes	Yes	+	Moderate	May synergistically improve clearance with other strategies	Complex device, sustained infusion and removal of excess ions needed, risk of ion (sodium) loading
Changing pH	Modification of PBUT–protein binding	2–3	No	No	+/−	High	Non-pharmacological approach	No effect in (patho-)physiological window, requires precise monitoring
Electromagnetic Waves (EM)	Modification of PBUT–protein binding	1–3	No	No	+	Moderate	Non-invasive, easily reversible	Strong EM emissions may interfere with other devices
Sorbents in Dialysis Membrane	Adsorption in membrane	4	No	No	+	Low	Easily integrated into conventional HD	Complex manufacturing
Sorbents in Dialysate	Direct adsorption from dialysate	4	No	No	+	Low	Easily integrated in conventional HD, no direct blood contact	Sustained infusion or sorbent regeneration needed
Fractionated Plasma Adsorption (FPAD)	Direct adsorption from fractioned plasma	4–6	Yes	Yes	++	Moderate	No direct blood cell contact	Complex device, costly
Hemoperfusion Cartridges	Direct adsorption from blood by porous beads	8–9	Yes	Yes	+/−	Low	Already available	Uncertain efficacy
Bioartificial Kidney (BAK)	Active tubular secretion by PTECs	3–5	Yes (not for PBUT removal)	Yes (not for PBUT removal)	+++	Moderate	Mimics native kidney function with potentially additional benefits	Device scale-up and manufacturing scalability hurdles, cost-effectiveness uncertain, regulatory challenges

In addition to dialytic removal strategies, the gastrointestinal tract plays a central role in the generation and absorption of PBUT precursors. Interventions targeting intestinal transit, microbiota composition, or intestinal binding of PBUT precursors have therefore been proposed as complementary strategies to reduce systemic PBUT burden. As these concepts fall beyond the scope of this review, which focuses on dialytic removal, readers are referred to dedicated reviews for further information [8,138].

In conclusion, while currently available dialysis modalities/schemes such as HDF and extended HD do not seem to have a clinically relevant impact on plasma PBUT concentrations in the long term, several novel techniques at varying stages of development show potential for future clinical use to enhance PBUT clearance. The efficacy of such treatments needs clinical validation, and the effects of a sustained reduction in plasma PBUT concentrations on hard endpoints such as morbidity and mortality should be examined to determine the added value of PBUT-lowering strategies.

## Data Availability

No new data were created or analyzed in this study.

## Supplementary Materials

The following supporting information can be downloaded at: https://www.mdpi.com/article/10.3390/toxins18010057/s1, Table S1: HDF; Table S2: Displacers, pH and ionic strength; Table S3: Adsorbents.

## Abbreviations

The following abbreviations are used in this manuscript:

AC	Activated carbon
BAK	Bioartificial kidney
BCRP	Breast cancer resistance protein
BSA	Bovine serum albumin
ciPTEC	Conditionally immortalized PTEC
CMPF	3-carboxy-4-methyl-5-propyl-2-furanpropionic acid
CRRT	Continuous renal replacement therapy
DHA	Docosahexaenoic acid
EM	Electromagnetic
ESKD	End-stage kidney disease
FFA	Free fatty acid
FPAD	Fractionated plasma separation, adsorption, and dialysis
HA	Hippuric acid
HD	Hemodialysis
HDF	Hemodiafiltration
HFR	Hemofiltration–reinfusion
HSA	Human serum albumin
IAA	Indole-3-acetic acid
IPIS	Increased plasma ionic strength
IS	Indoxyl sulfate
KA	Kynurenic acid
LDH	Lactate dehydrogenase
MMM	Mixed-matrix membrane
MRP	Multidrug resistance protein
NRF	Norfloxacin
OAT	Organic anion transporter
PBUT	Protein-bound uremic toxin
p-CG	P-cresyl glucuronide
p-CS	P-cresyl sulfate
pdHDF	Postdilution HDF
PTEC	Proximal tubular epithelial cell
PVP	Polyvinylpyrrolidone
RED	Rapid equilibrium dialysis
RKF	Residual kidney function
RR	Reduction ratio
TRL	Technology readiness level
TSR	Total solute removal
